# 211. Defining Optimal Sampling Times for Cefepime Therapeutic Drug Monitoring in Clinical Practice

**DOI:** 10.1093/ofid/ofae631.069

**Published:** 2025-01-29

**Authors:** Nathaniel J Rhodes, Brandon Smith, Ryan K Shields, Samantha Pan, Erin Weslander, Shannon Galvin, Jasmine Hughes, Maria-Stephanie Hughes, Fekade B Sime, Jason A Roberts, Patrick J Kiel, Michael N Neely, Marc H Scheetz

**Affiliations:** Midwestern University, Downers Grove, IL; University of Pittsburgh Medical Center, Pittsburgh, Pennsylvania; University of Pittsburgh, Pittsburgh, Pennsylvania; Northwestern Memorial Hospital, Chicago, IL; Northwestern Memorial Hospital, Chicago, IL; Northwestern University Feinberg School of Medicine, Chicago, Illinois; InsightRX, San Francisco, California; InsightRX, San Francisco, California; Centre for Translational Anti-infective Pharmacodynamics, UQ Centre for Clinical Research, Brisbane, Queensland, Australia; UQ Centre for Clinical Research, Faculty of Medicine, Brisbane, Queensland, Australia; Indiana University School of Medicine, Indianapolis, Indiana; The Saban Research Institute, Children’s Hospital Los Angeles, University of Southern California, Los Angeles, CA, USA, Los Angeles, California; Midwestern University, Downers Grove, IL

## Abstract

**Background:**

Clinicians performing beta-lactam therapeutic drug monitoring (TDM) lack evidence on when levels should ideally be drawn after a dose. Herein, we define the optimal timing (i.e., optimal sampling) for cefepime using real-world TDM data to validate our approach.

**Figure 1.**
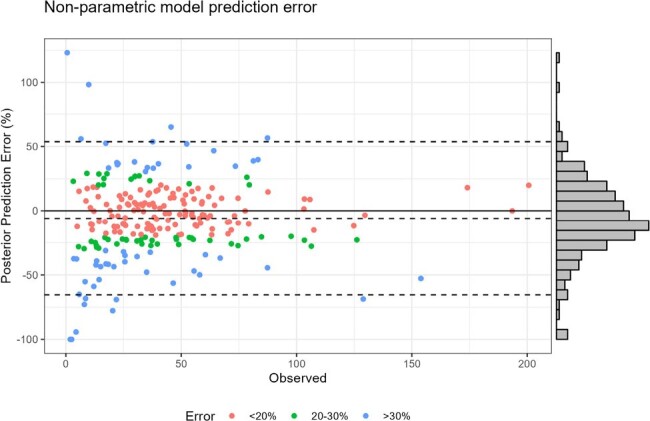
External validation of the Bayesian prior non-parametric population PK model

**Methods:**

De-identified data from two centers performing routine cefepime TDM were extracted by InsightRX and served as an external validation cohort. Plasma cefepime was quantified using validated LC-MS/MS assays for TDM and dosing was protocolized at each site. CRRT and ECMO patients were included but other dialysis patients were not. Bias (MPE) and precision (RMSE) of a non-parametric prior were assessed. Multiple-model optimal (MM-opt) sampling strategies were estimated for the first 24 hours of treatment. To mirror clinical practice, one- and two-sample designs were evaluated. Dose and covariate values informed optimal sampling times. Bayesian PK exposures were compared using all samples, trough-only sampling, or using a single optimally timed sample. AUCs were calculated from the posteriors. For *f*T _>MIC_ analysis, the MIC was fixed at 8 mg/L. We used Pmetrics 2.1.1 for R.

**Figure 2.**
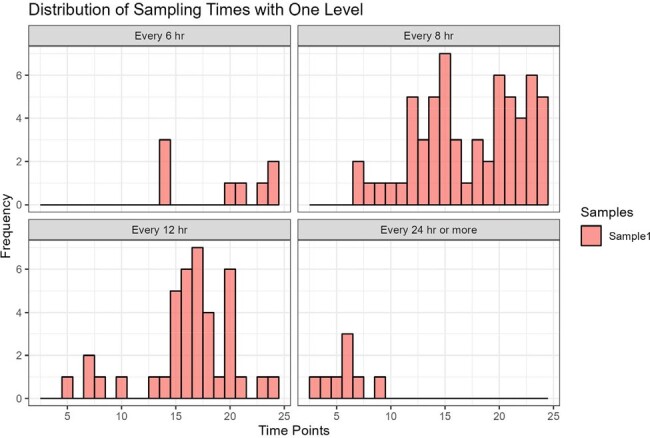
Distribution of MM-optimal sampling times in external validation data using n=1 sample

**Results:**

116 patients (42% female; median age, CRCL, and weight: 62 years, 76 mL/min, and 80 kg, respectively) contributed 235 levels. The PK model demonstrated acceptable bias and precision (-6% MPE, 30.9 RMSE) as a prior for estimating exposures from the TDM data (Fig1). For a one-sample approach, the most common MM-opt sampling times varied (Fig2) but were often a mid-point or trough. In the two-sample approach, sample one was often a mid-point and sample two was often a trough (Fig3). First 24-hr AUC and *f*T_>MIC_ did not significantly differ using all available samples for analysis vs. limiting sampling to a single optimized time point vs. limiting sampling to a trough-only approach (P >0.05 for all comparisons; Fig4).

**Figure 3.**
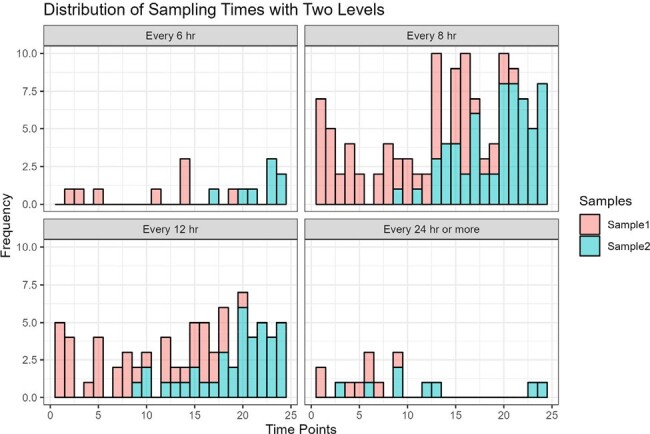
Distribution of MM-optimal sampling times in external validation data using n=2 samples

**Conclusion:**

Optimal cefepime sampling times depended on dosing regimen, and renal disposition. When limited to a single sample, optimal sampling times for cefepime TDM were often midpoint/trough levels, but when two samples were obtained the optimal sampling times were often a mid-point followed by a trough. Estimation of PK and PK/PD exposures was not significantly worse when using a validated Bayesian prior and a trough-only sampling approach.

**Figure 4.**
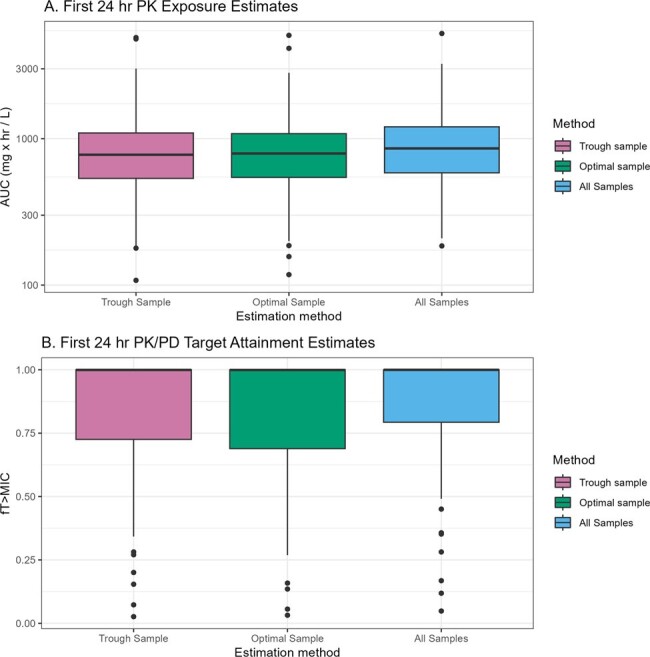
First 24 hours PK and PK/PD exposure estimates from Bayesian analysis of all external validation data and Bayesian analysis of n=1 optimally timed samples from the same population

**Disclosures:**

**Nathaniel J. Rhodes, PharmD MS**, Apothecademy, LLC: Advisor/Consultant **Brandon Smith, MD, PharmD**, Melinta Therapeutics: Advisor/Consultant|Shionogi, INC: Advisor/Consultant **Ryan K. Shields, PharmD, MS**, Allergan: Advisor/Consultant|Cidara: Advisor/Consultant|Entasis: Advisor/Consultant|GSK: Advisor/Consultant|Melinta: Advisor/Consultant|Melinta: Grant/Research Support|Menarini: Advisor/Consultant|Merck: Advisor/Consultant|Merck: Grant/Research Support|Pfizer: Advisor/Consultant|Roche: Grant/Research Support|Shionogi: Advisor/Consultant|Shionogi: Grant/Research Support|Utility: Advisor/Consultant|Venatorx: Advisor/Consultant|Venatorx: Grant/Research Support **Jasmine Hughes, PhD**, InsightRX: Employee|InsightRX: Stocks/Bonds (Private Company) **Maria-Stephanie Hughes, PharmD**, InsightRX: Employee of company|InsightRX: Stocks/Bonds (Private Company) **Fekade B. Sime, PhD, S.Aust.**, Gilead: Grant/Research Support|Pfizer: Grant/Research Support **Patrick J. Kiel, PharmD**, Amgen: Employee|Amgen: Stocks/Bonds (Public Company) **Marc H. Scheetz, PharmD, MSc**, Abbvie: Advisor/Consultant|Basilea: Advisor/Consultant|Cidara: Advisor/Consultant|DoseMe: Advisor/Consultant|Entasis: Advisor/Consultant|F2G: Advisor/Consultant|GSK: Advisor/Consultant|Lykos: Advisor/Consultant|Roche: Advisor/Consultant|Third Pole Therapeutics: Advisor/Consultant|Xelia: Advisor/Consultant

